# Design of a MEMS-Based Oscillator Using 180nm CMOS Technology

**DOI:** 10.1371/journal.pone.0158954

**Published:** 2016-07-08

**Authors:** Sukanta Roy, Harikrishnan Ramiah, Ahmed Wasif Reza, Chee Cheow Lim, Eloi Marigo Ferrer

**Affiliations:** 1 Department of Electrical Engineering, University of Malaya, 50603 Kuala Lumpur, Federal Territory of Kuala Lumpur, Malaysia; 2 Silterra Malaysia Sdn. Bhd.(368948-D), 47800 Petaling Jaya, Selangor, Malaysia; Lanzhou University of Technology, CHINA

## Abstract

Micro-electro mechanical system (MEMS) based oscillators are revolutionizing the timing industry as a cost effective solution, enhanced with more features, superior performance and better reliability. The design of a sustaining amplifier was triggered primarily to replenish MEMS resonator’s high motion losses due to the possibility of their ‘system-on-chip’ integrated circuit solution. The design of a sustaining amplifier observing high gain and adequate phase shift for an electrostatic clamp-clamp (C-C) beam MEMS resonator, involves the use of an 180nm CMOS process with an unloaded *Q* of 1000 in realizing a fixed frequency oscillator. A net 122dBΩ transimpedance gain with adequate phase shift has ensured 17.22MHz resonant frequency oscillation with a layout area consumption of 0.121 mm^2^ in the integrated chip solution, the sustaining amplifier draws 6.3mW with a respective phase noise of -84dBc/Hz at 1kHz offset is achieved within a noise floor of -103dB_C_/Hz. In this work, a comparison is drawn among similar design studies on the basis of a defined figure of merit (FOM). A low phase noise of 1kHz, high figure of merit and the smaller size of the chip has accredited to the design’s applicability towards in the implementation of a clock generative integrated circuit. In addition to that, this complete silicon based MEMS oscillator in a monolithic solution has offered a cost effective solution for industrial or biomedical electronic applications.

## Introduction

Reference frequency sources typically generate a single frequency ranging from 1MHz to 50MHz for the use of a channel rate and multi clock domain phase locked loops (PLL), which synthesizes an output ranging from lower megahertz to over 1GHz. Present electronic devices, such as personal computers, servers and embedded systems are in need of timing clocks to achieve synchronization of operation. However, frequency accuracy is flexed in some data interface protocols, such as HS-USB, S-ATA and 10/11/1000 Ethernet, where higher ppm is in need. For example, reference oscillator in USB 2000 maintains ±500ppm, serial ATA 2005 needs ±350 and IEEE Std. 1998 needs ±100ppm [1–3].

In the past, bulky off-chip quartz based ICs were the preferred choice for reference timing generation. Comparatively, MEMS electrostatic resonator based oscillator proved its significance with its system-on-chip (SoC) solution where an inductorless design proved an excellent supplementary choice for the RC-VCO, complying with OC-48 application criteria [4, 5]. Nevertheless, the electrostatic resonator based oscillator lags with some setbacks in some dedicatedapplications, but exhibits promising performances in the application of real time clocking (RTC) with a comparatively low output signal level in the MEMS based oscillator, due to the high motional losses of the resonator. The RTC adaptation was deemed appropriate as low signal levels and jitter/noise pattern exhibits no major concern in the clocking application of [6–8]. Potential biomedical application of portable optical needle substitution by CMOS circuitry [9], is an example to be cited in this work, as the RTC employed chip technology can perform on par with the previously used bulky equipment, to offer logical functions needed for the optical resolution of tissue planes. At its strongest point, clock IC’s are desirably miniaturized through the implementation of MEMS device, as its favorable small form factor edges over its quartz counterpart.

Two distinct resonator actuations for MEMS structures are piezoelectric and capacitive. The piezoelectric actuation naturally exhibits much lower motional resistance and a higher power handling capability than the actuation in electrostatic resonator [10, 11]. In the view of SoC implementation, onto low cost silicon substrate, capacitive-based electrostatic resonators leads in preference [12–14]. As such, the sustaining amplifier is loaded with the *Q* factor of the resonator in the oscillator. The capacitive-based resonator limits the operation bandwidth of the transimpedance amplifier (TIA), thus limiting the oscillation range. This work depicts the design and implementation of CMOS based single ended sustaining amplifier in sustaining the oscillation of a MEMS resonator. The objective of the work is to deliver a highly competitive performance oscillator through the optimization of phase noise and power dissipation in the application of clock generation IC. A comparatively low cost, temperature uncompensated Si clamp-clamp beam resonator is chosen for a much higher figure of merit realization of the complete electrostatic oscillator. For the oscillator, the operation frequency is 17.22MHz with a 95% yield of frequency in 180nm CMOS process implementation. Now, section 2 of this paper reports the descriptive characteristic of the MEMS resonator in MHz application, followed by the review on the design of the sustaining amplifier in Section 3. Section 4 summarizes the experimental and simulation results obtained. It also encapsulates a discussion by comparing the performance of the proposed work with other similar works and a process corner sustainability study of the post layout design through Monte Carlo simulation tools. Lastly, the conclusion of this work is given in Section 5.

## Electrostatic MEMS Resonator

The operations of MEMS sensing can be realized as common base, resistive or capacitive detection [15]. The common base detection and resistive detection, exhibits higher noise effect compared to its counterpart, of capacitive detection. Common base detection has an input of current noise which is proportional to the increase of the transconductance of the input MOS, whereas in the resistive detection amplifier, noise is dominated by the conversion resistance, resulting in reduced bandwidth of operation [16]. Since noise seems to be the governing performance factor in the intended application of clock ICs, capacitive detection is deemed to be the least noisiest is avoided in appreciating the degrading parasitic effects resulting to the reduction in bandwidth [17, 18].

In MEMS devices, air damping, thermos-elastic damping and acoustic energy loss in anchors are contributing sources of mechanical loss. Although vacuum packaging is used to suppress air damping in present C-C MEMS, the major contribution of loss is due to the periodic wave to the anchor.

## Design of Sustaining Amplifier

In sustaining the oscillation, an integrating amplifier is essential with a positive gain and a sufficient phase shift [15]. In this work, a CMOS based single ended-single rail amplifier is integrated as the sustaining amplifier circuit. The circuit desirably inherits sufficient gain-bandwidth in the feedback compensation. The architecture is targeted to exhibit competitive phase noise, with minimal DC power dissipation, leading towards a promising Figure of Merit (FOM) with an acceptable output voltage swing.

In this work, the sustaining amplifier consists of a transimpedance amplifier (TIA), followed by a non-inverting voltage amplifier and subsequently a cascode stage. The amplifier is terminated with a 50Ω output buffer for characterization purposes. The ‘capacitively read’ motional current is fed into the TIA and reintroduced back to electrostatically excite the resonator. A topology based feedback analysis technique can be interpreted in deriving the gain and output resistances for each particular stage of this sustaining amplifier which gets support from [19], [20] and [21].

### Design of transimpedance amplifier stage

**Fig 1:**
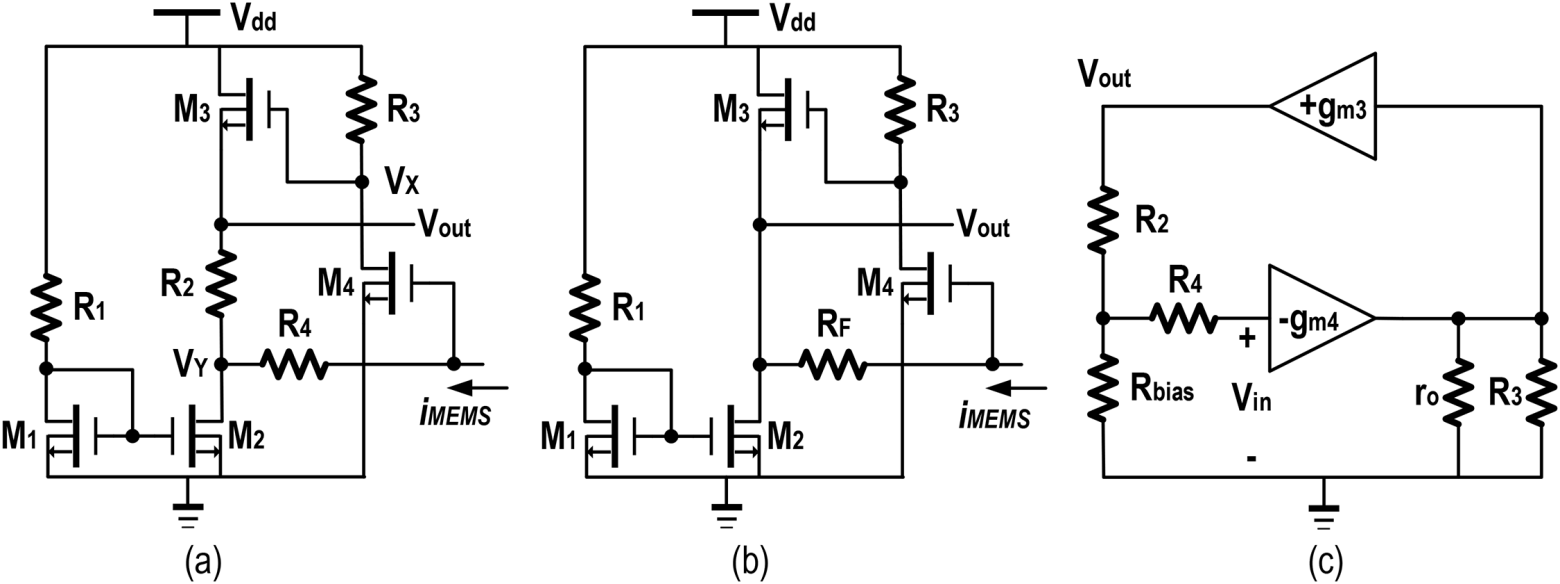
Transimpedance amplifier schematic diagram along with bias circuitry. a) Feedback resistor being divided into *R*_*2*_ and *R*_*4*_ for enhancing linearity b) Equivalent feedback resistor *R*_*F*_ c) The small signal equivalent model.

The C-C beam resonator operates in electrostatic capacitive transduction mode, generates a small alternating current signal in its read-out electrode. In this work, a regulated cascode (RGC), shunt-shunt feedback TIA is developed, where the feedback resistor has been divided into two equal parts to ensure better linearity. Fig 1 shows the schematic of the TIA, which inherits high gain, high linearity and low noise performance. Fig 1 illustrates, the core of the amplifier consists of a common source stage and a source follower stage. The source follower isolates the resistor, *R*_*3*_ from the loading effect of the feedback resistors *R*_*2*_ and *R*_*4*_. The closed loop gain is made adjustable by *R*_*2*_ and *R*_*4*_, in a pre-condition that the output impedance of the source follower is kept much lesser than the feedback resistor.

When current from MEMS is zero, then the gate voltage of M_4_ is equal to the DC value of output voltage, *v*_*out*_. As *i*_*MEMS*_ increases, most of the current will flow through *R*_*4*_ and sink via the current source. Along with this, the current flow through M_3_ and *R*_*2*_ will also be increased in which, the MEMS output current is converted and sensed as a voltage drop signal across *R*_*2*_. However, for a substantial increase in *i*_*MEMS*_, the voltage drop across *R*_*4*_ will reduce *v*_*out*_ and the current source is driven into the linear region of operation, hence sacrificing the linearity. *R*_*4*_ is designed to have a minimum value in maintaining the linearity, whereas *R*_*2*_ is adjusted for high gain/noise performance.

Fig 1 illustrates the working principle of the TIA. In reference to Fig 1(a), the output voltage, *V*_*out*_ is sensed and its proportional current is routed through *R*_*2*_ back to the summing point, V_Y_ at its input. At this point, V_Y_ shunt-shunt feedback is actuated and simultaneously linearity is enhanced by dividing *R*_*F*_ into *R*_*2*_ and *R*_*4*_. The gain expression developed in reference to Fig 1(b), is given as in Eq (1).

$$V_{in}-V_{out}=I_{RF}\times R_{F}$$ (1)

Considering, *I*_*bias*_ = (*I*_*MEMS*_ + *I*_*R2*_) and *I*_*RF*_ = *I*_*MEMS*_ = *I*_*R4*_, the voltage *V*_*x*_ at the node of *R*_*3*_ can be written as *V*_*x*_ = —(*g*_*m3*_ × *R*_*3*_ × *V*_*in*_) = *V*_*out*_, from which the open loop gain is obtained.

$$\frac{V_{out}}{V_{in}}=-g_{m3}\times R_{3}$$ (2)

Substituting Eq (2) into Eqs (1) and (3) can be yielded.

$$\frac{V_{out}}{I_{MEMS}}=\frac{-R_{F}(g_{m3}R_{3})}{1+g_{m3}R_{3}}$$ (3)

With the incorporation of *R*_*2*_ and *R*_*4*_ in increasing its stability, the output voltage is derived hereby.

Vin−Vout=IMEMS×R4×IR2×R2⇒Vout(1−gm3R3−1)=IMEMS(R4−R2)+IRR32⇒Vout=IMEMS((−gm3R3)(R4−R2)1+gm3R3)+IR3((−gm3R3)(R2)1+gm3R3) (4)

By comparing Eq (3) with Eq (4), it is evident that, in the resistive feedback configuration, when *I*_*MEMS*_ or *V*_*in*_ is increased, the slope of Eq (4) decreases. With this increased linearity and better stability, the close loop transimpedance gain, *R*_*T*_ can be derived. In considering a larger aspect ratio of M_3_, it can be deduced that, (1/*g*_*m3*_) << (*R*_*2*_ + *R*_*4*_) for which the output impedance referring into the source follower (consists of M_3_ and current source) approximates to 1/*g*_*m3*_. Thus, the close loop transimpedance gain, defined by *R*_*T*_ becomes the sum of the adjustable resistances in the feedback loop as seen in Eq (5), in the event that the open loop gain of Eq (2) is much greater than unity.

$$R_{T}=\frac{g_{m3}R_{3}}{1+g_{m3}R_{3}}\times \left(R_{4}+R_{2}\right)\approx \left(R_{4}+R_{2}\right)$$ (5)

### Design of an intermediate stage voltage amplifier

Subsequently, after the read-out signal is acquired as the capacitive current from the MEMS resonator and converted into a voltage signal by the TIA, the voltage amplifier follows with enhanced gain and extended bandwidth operation. There is a necessity of incorporating adequate phase shift from the input to output in sustaining the oscillation. In this work, a modified Cherry-Hooper voltage amplifier topology is designed for a nominal gain with zero phase shifts and an extended bandwidth. This block is designed to compensate the amount of gain, which is necessary to replenish the decrease of loop gain due to the relatively high load capacitance corresponding to the inputs of the integrating output buffers [22].

**Fig 2:**
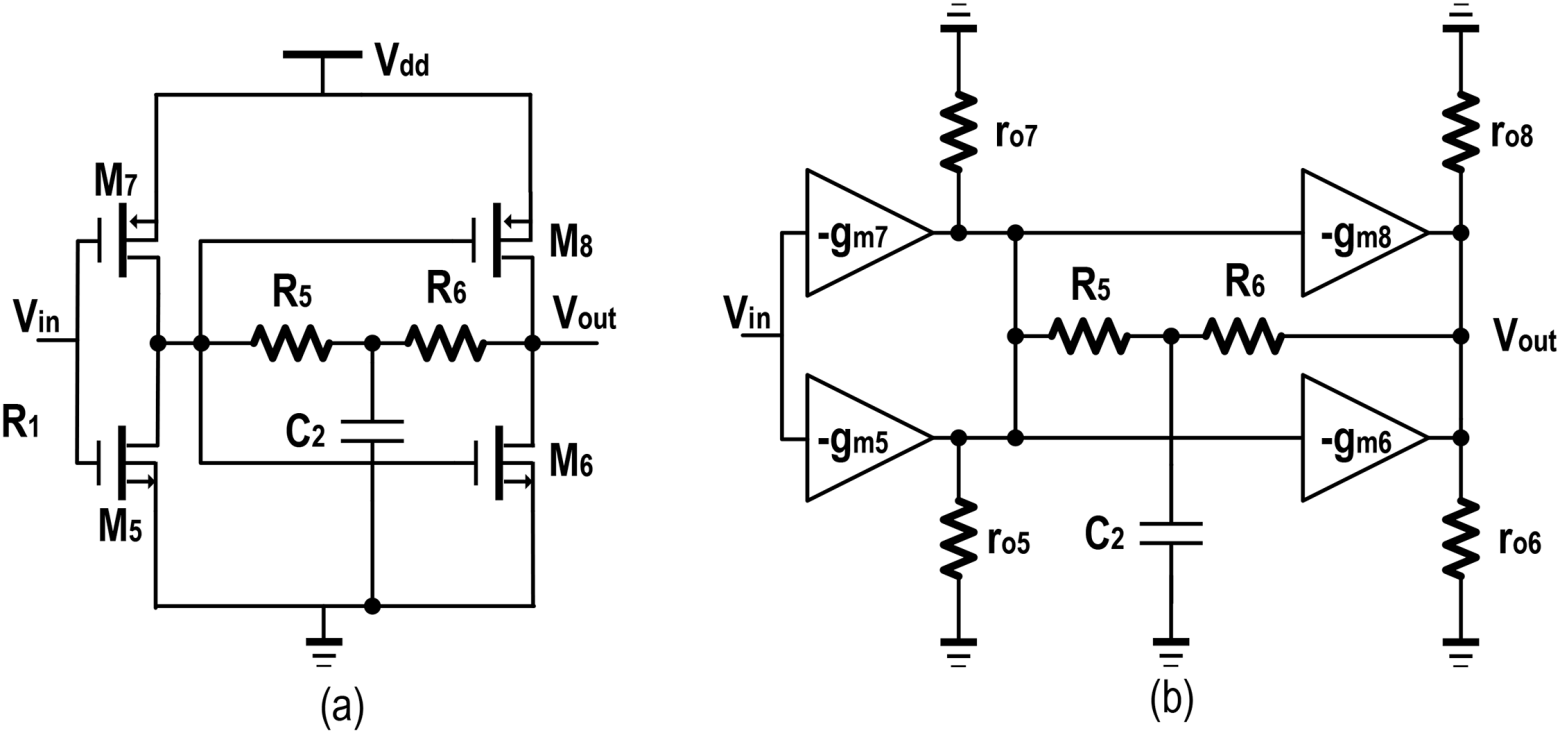
Intermediate voltage amplifier. a) Schematic diagram b) The small signal equivalent model.

The schematic diagram of the voltage amplifier is shown in Fig 2. The push-pull inverter topology is chosen in preference for its simplicity and to enhance the output with less distortion per active device [23]. This second stage is the gain tuning stage achievable with the integration *R*_*6*_-*C*_*2*_ ‘T’ network. This ‘T’ network in this shunt-shunt feedback configuration would introduce an extra zero, which will be used for pole cancellation in Eq (8) and offers less input and output impedance in subsequent stages of integration. As this *R*_*6*_-*C*_*2*_ would favorably shift the poles to higher frequency, the bandwidth will also be increased. As observed from the small signal equivalent circuit, the expression of total gain could be derived for two back to back push pull amplifier circuits where the gain of each stage can be derived to be combined finally.

$$\frac{v_{x}}{v_{in}}=(g_{m5}+g_{m7})(r_{05}||r_{07})$$ (6)

$$v_{R5}=(v_{out}-v_{x})=(v_{x})(\frac{sR_{5}C_{2}}{1+sR_{5}C_{2}})$$ (7)

Combining Eqs (6) and (7) while eliminating for *v*_*x*_, the final voltage gain is obtained in Eq (8).

$$\frac{v_{out}}{v_{in}}=\frac{\left(g_{m5}+g_{m7})(r_{05}||r_{07})(1+s2R_{5}C_{2}\right)}{1+sR_{5}C_{2}}$$ (8)

From Fig 2(b), the output impedance can be written as shown in Eq (9).

$$z_{out}\approx \frac{\left(r_{o6}||r_{o8}\right)R_{6}\left[R_{5}{}^{2}+R_{6}\left(R_{5}+r_{o5}||r_{o7}\right)\left(R_{5}C_{2}s+1\right)\right]}{\left(R_{6}+r_{o6}||r_{o8}\right)\left[R_{5}{}^{2}+R_{6}\left(R_{5}+r_{o5}||r_{o7}\right)\left(R_{5}C_{2}s+1\right)\right]+R_{5}\left(r_{o6}||r_{o8}\right)\left[g_{m6}R_{6}\left(r_{o5}||r_{o7}\right)-R_{5}+r_{o5}||r_{o7}\right]}$$ (9)

Capacitance, *C*_*2*_ in Eq (9) will ensure a complete pole cancellation in extending the operational bandwidth. In this design, the transconductance of M_6_ is kept much higher than the feedback resistance (*R*_*5*_ +*R*_*6*_) to avoid the phase shift resulting from the resistive loss. For a designated frequency ω_*T*_ in Eq (10), for which the pole would be cancelled, Eq (9) could be further simplified to acquire the desired output impedance given in Eq (11).

$$\left|\omega_{T}\right|\approx \frac{R_{6}+R_{5}}{R_{6}R_{5}C_{2}}$$ (10)

$$\begin{array}{l} \begin{array}{r} z_{out}\approx \frac{\left(r_{o6}||r_{o8}\right)\left[(R_{5}+R_{6})+\left(r_{o5}||r_{o7}\right)\right]}{\left(r_{o6}||r_{o8}\right)\left[1+g_{m6}\left(r_{o5}||r_{o7}\right)+\right]R_{5}+(r_{o5}||r_{o7})}\\ \Rightarrow z_{out}\approx \frac{(R_{5}+R_{6})+\left(r_{o5}||r_{o7}\right)}{g_{m6}\left(r_{o5}||r_{o7}\right)}\approx \frac{1}{g_{m6}} \end{array} \end{array}$$ (11)

The value of *R*_*5*_ and the aspect ratio of M_6_ are tuned to replenish the gain, thus driving the targeted MEMS resonator. Also, as for its inverted buffer structure which consists of M_5_, M_6_, M_7_ and M_8_, it ensures a near zero phase shift over the bandwidth of interest at 17.22 MHz of operation. This phase shift is necessary for sustaining the amplifier block, designed to comply the Barkhausen criterion.

### Design of Cascode voltage amplifier stage

In the third stage of this single ended sustaining amplifier block, a cascode amplifier is integrated reflecting to additional voltage gain with a proportional phase shift. Cascode stage or the combination of common source (CS) and common gate (CG) amplifier is a good choice for high gain, with moderate input impedance and for an overall improved bandwidth operation. This configuration is deemed to be further stable as its output is electrically and physically isolated from the input.

**Fig 3:**
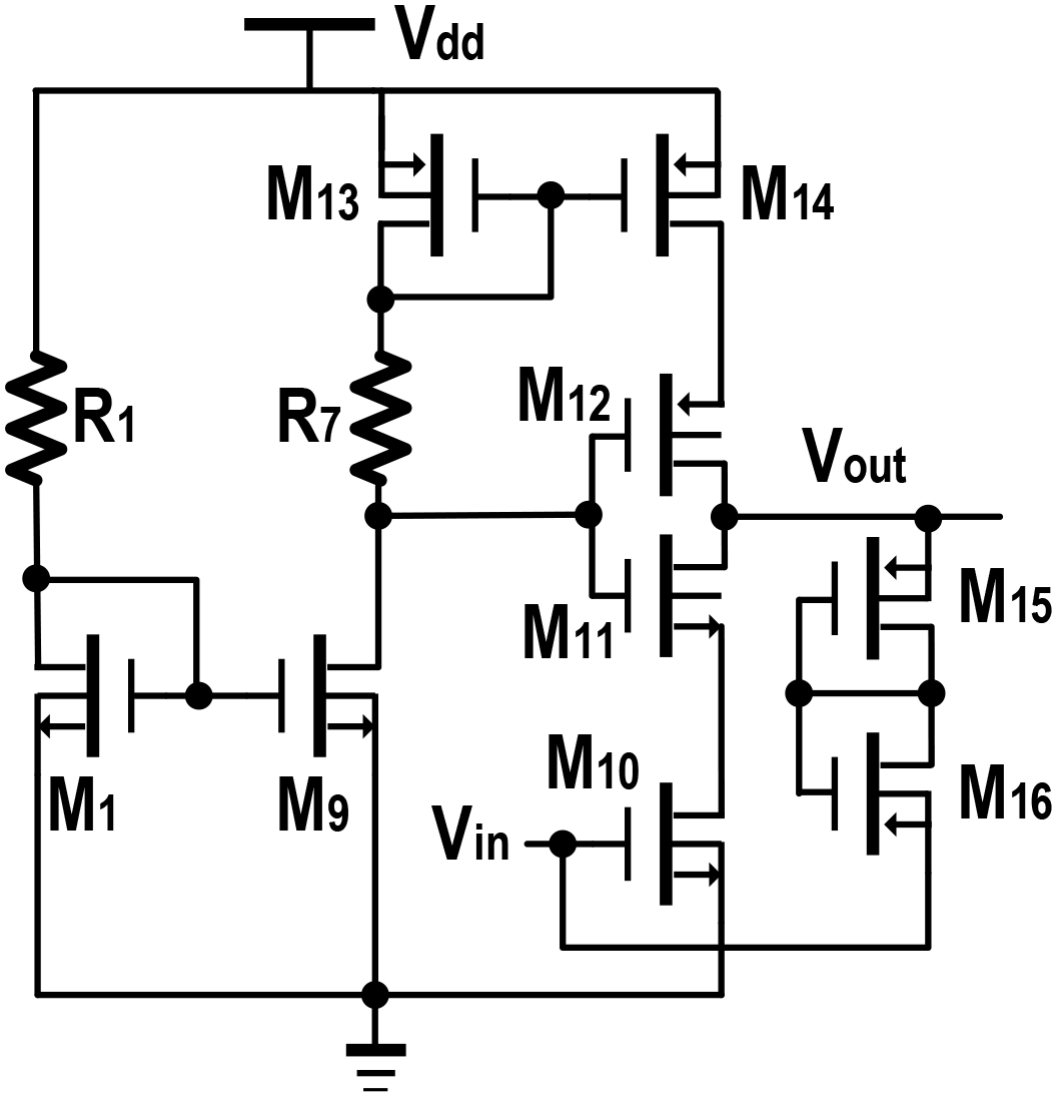
Cascode voltage amplifier with integrated bias circuitry consists of M_1_, M_9_, M_13_, M_14_, *R*_*7*_ and *R*_*1*._

**Fig 4:**
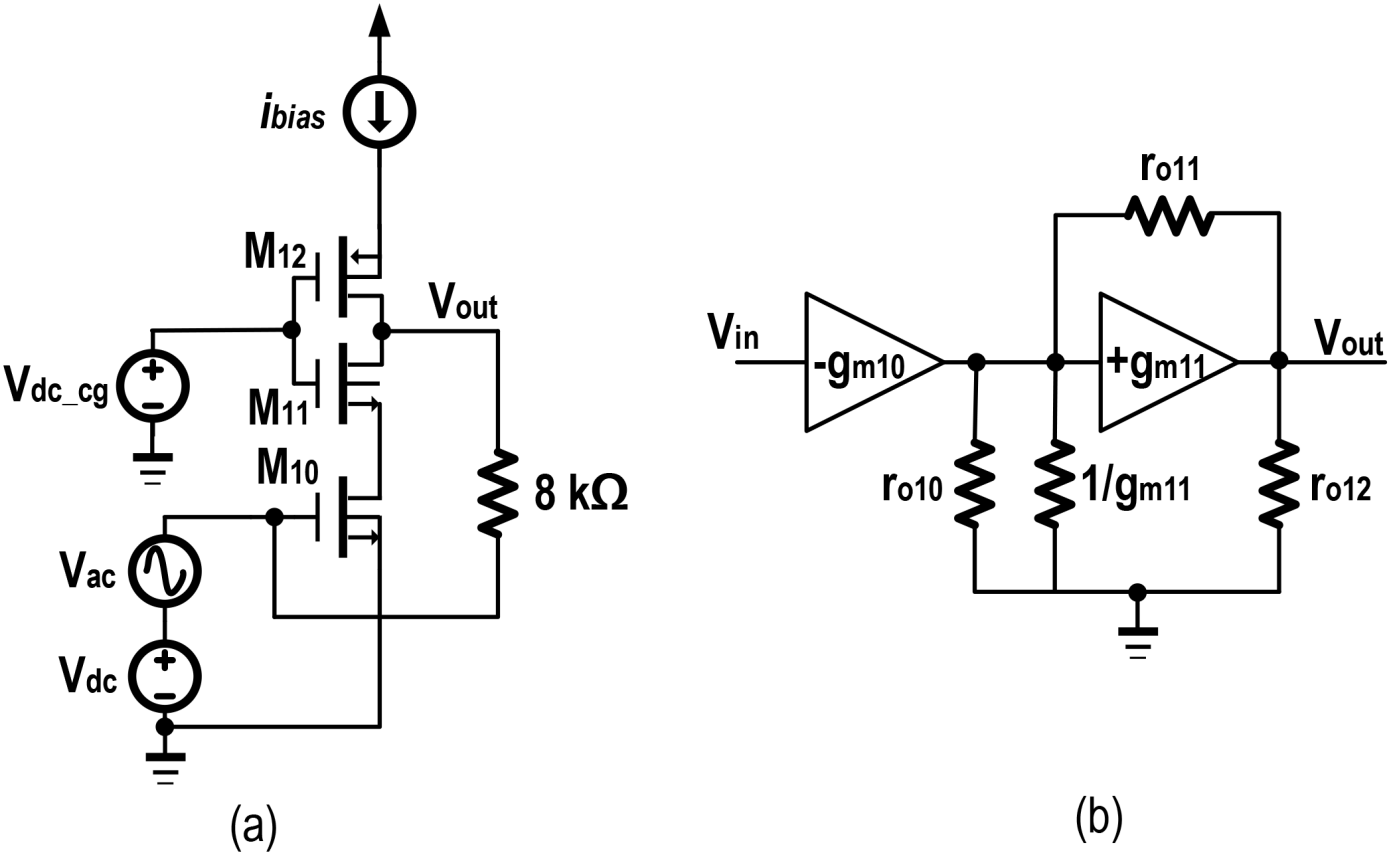
Cascode voltage amplifier. a) Simplified schematic diagram with 8k8Ω resistances b) The small signal equivalent model.

In Fig 3, the CS amplifier is denoted with M_10_, whereas M_11_ behaves as a CG stage. The feedback path from the output node to the gate of CS is through high resistance (~8k8Ω), which is implemented by M_15_ and M_16_, in a PMOS pseudo-resistor configuration. This feedback bias the CS stage (M_10_), hence the output voltage signal from the previous stage of Fig 2, will assist this biasing through a 6pF decoupling capacitor, C_1_ (not shown). Moreover, M_12_ gives an additional gain depending on the bias point of the CG stage. The current source consists of M_13_, M_14_ and *R*_*7*_ along with M_9_ ensures the necessary biasing for CG stage. Fig 4(a), reflects a simplified representation of the cascode stage from which the small signal equivalent circuit is constructed in Fig 4(b). Referring to the nodes *x* and *v*_*out*_, Eqs (12) and (13) can be derived.

$$(g_{o10}+g_{o11}+g_{m11})\times v_{x}-g_{o11}\times v_{out}=-g_{m10}\times v_{in}$$ (12)

$$(-g_{m11}-g_{o11})\times v_{x}+(g_{o11}+g_{o12})\times v_{out}=0$$ (13)

Solving Eqs (12) and (13), the desired gain expression is given as in Eq (14).

$$\frac{v_{out}}{v_{in}}=\frac{-g_{m10}\left(g_{o11}+g_{m11}\right)}{g_{o10}g_{o11}+g_{o10}g_{o12}+g_{o11}g_{o12}+g_{o12}g_{m11}}≅\frac{-g_{m10}}{g_{o12}}\equiv -g_{m10}\times r_{o12}$$ (14)

Similarly, the output impedance can be found to be approximately equal to *r*_*o12*_ given by Eq (15).

$$r_{out}=\left[r_{o10}+r_{o11}+\left(g_{m11}r_{o10}r_{o11}\right)\right]||\left(r_{o12}\right)\approx r_{o12}$$ (15)

## Results and Discussion

In characterizing the design of the sustaining amplifier and further completing the oscillator architecture, several verification analyses have been done on a 0.18μm CMOS platform through the use of Cadence Spectre. Open loop responses are necessary to preliminarily determine the ability of the amplifier in compensating the motional losses which can be estimated from the resonator’s forward transmission responses, S_21_. Once the oscillation is configured to sustain via the Barkhausen criterion [3, 24, 25], then a close loop analysis for the whole resonator is discussed to measure the design’s competence in a clock generator application with regards to frequency stability, phase noises, noise floor, etc.

### MEMS characterization and parameters extraction

**Fig 5:**
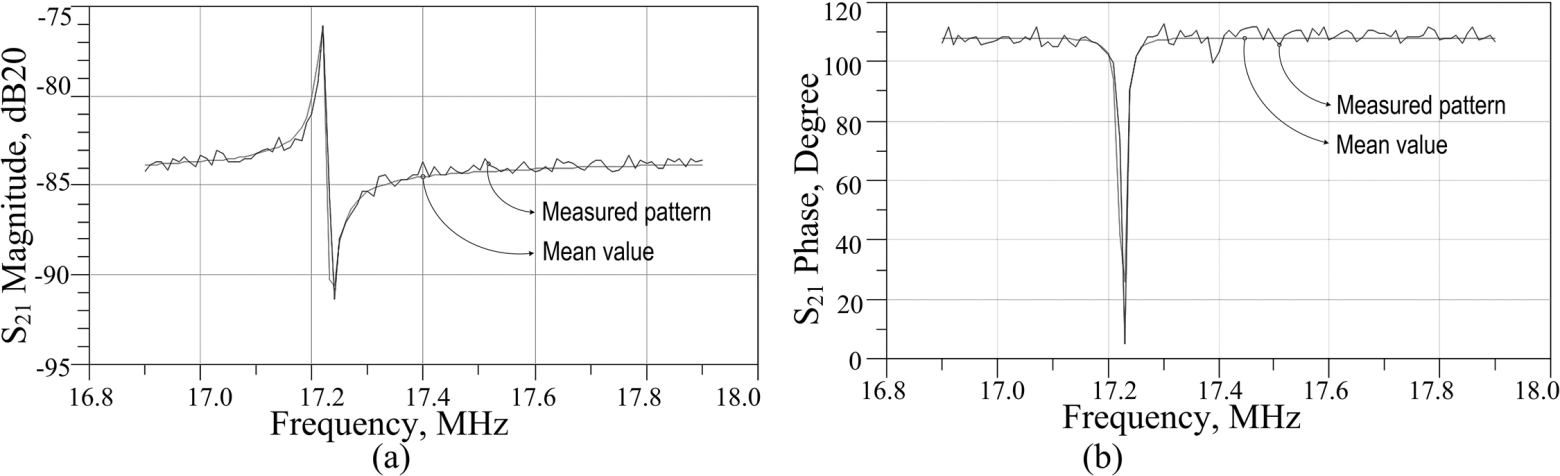
Resonator’s behavioral response extracted by a spectrum analyzer. a) S_21_ magnitude plot b) S_21_ phase plot.

The referred C-C beam MEMS resonator is verified for its insertion loss and total phase shift estimation. In extracting the result, an experimental setup is constructed in extracting the scattering parameter at the excited state of the resonator with an external DC bias of 5V. From this spectrum of data, the resonant frequency is observed at 17.22MHz. Other physical parameters, such as beam width, beam length, air gap, poisons co-efficient, and Young’s modulus are used along with the resonant frequency to determine the electrical series RLC parameters constructing this resonator. The extracted RLC values in the lumped component model are verified from its spectrum analysis where it matches with the measured data, as described in Fig 5.

**Fig 6:**
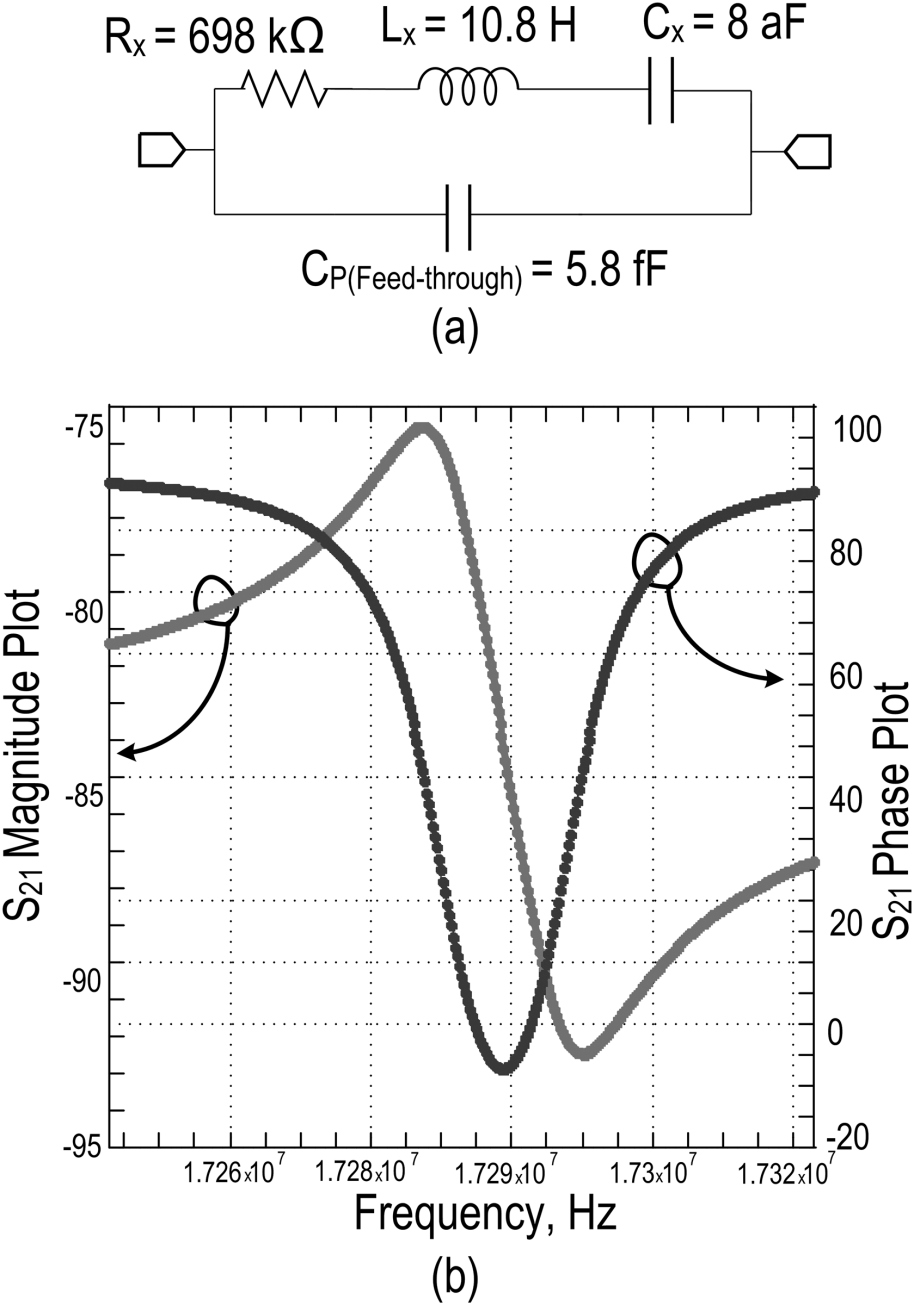
Simulation model of resonator. a) Electrical lumped parameter of RLC model b) S_21_ simulated response of lumped parameter model.

In the model, the feed-through parasitic capacitance, C_P_ is approximated as 5.8fF by computing the capacitance from the overlap area between top and bottom electrodes. The edges are considered to be sharp enough for the overlap region and hence, the fringing field effect dominates. The value of C_P_ is found to be low from the geometry of the resonator and hence, it is assumed that parallel resonance does not force the resonator into non-linear vibration. The negligible deviation seen in the responses of Fig 5 are from unavoidable parasitic capacitances and resistances in real measurement apparatus, such as in the use of cable, ports, etc. The final component values for the two port electrical RLC model is shown in Fig 6 along with its simulated S-parameter analysis response. From this S_21_ response, an approximate 76dB of transmission loss can be found with a corresponding 95° phase shift. In Table 1, these findings are listed along with some main physical parameters of the MEMS device.

**Table 1:** Mechanical and Electrical Parameters for the C-C Beam Resonator.

	Parameter	Unit	Value
**Mechanical Data**	Effective stiffness, *k*	N/m	897
	Effective mass, *m*_*eff*_	Kg	76 f
	Unloaded quality factor, *Q*	~~~~	1000
	Transduction gap	m	90n
**Extracted and Measured Data**	Electromechanical coefficient, *k*_*eff*_^*2*^	%	0.461
	Series resonance frequency, *f*_*s*_ @ 5V_DC_	Hz	17.2631 M
	Parallel resonance frequency, *f*_*p*_@ 5V_DC_	Hz	17.30294 M
	Operating frequency,*f*@ 5V_DC_	Hz	17.22 M
	Electromechanical coupling factor, η	C/m	85 n
**Curve Fitted Electrical Data**	Motional resistance, *R*_*x*_	Ω	698 k
	Motional capacitance, *C*_*x*_	F	8 a
	Motional inductance, *L*_*x*_	H	10.6
	Feed-through capacitance, *C*_*p*_	F	5.8 f
	Insertion loss	dB	76
	Transmission phase	°	95

### Sustaining amplifier open loop response

Each block of the amplifier delivers a prescribed amount of gain with a respective phase shift. In an open loop AC analysis, the individual responses are verified across the bandwidth of operation in the absence of the resonator, in completing the loop. In substitute of a resonator, a small AC current source with 17.22MHz is added as an input to emulate the role of capacitive current output of the MEMS resonator.

**Fig 7:**
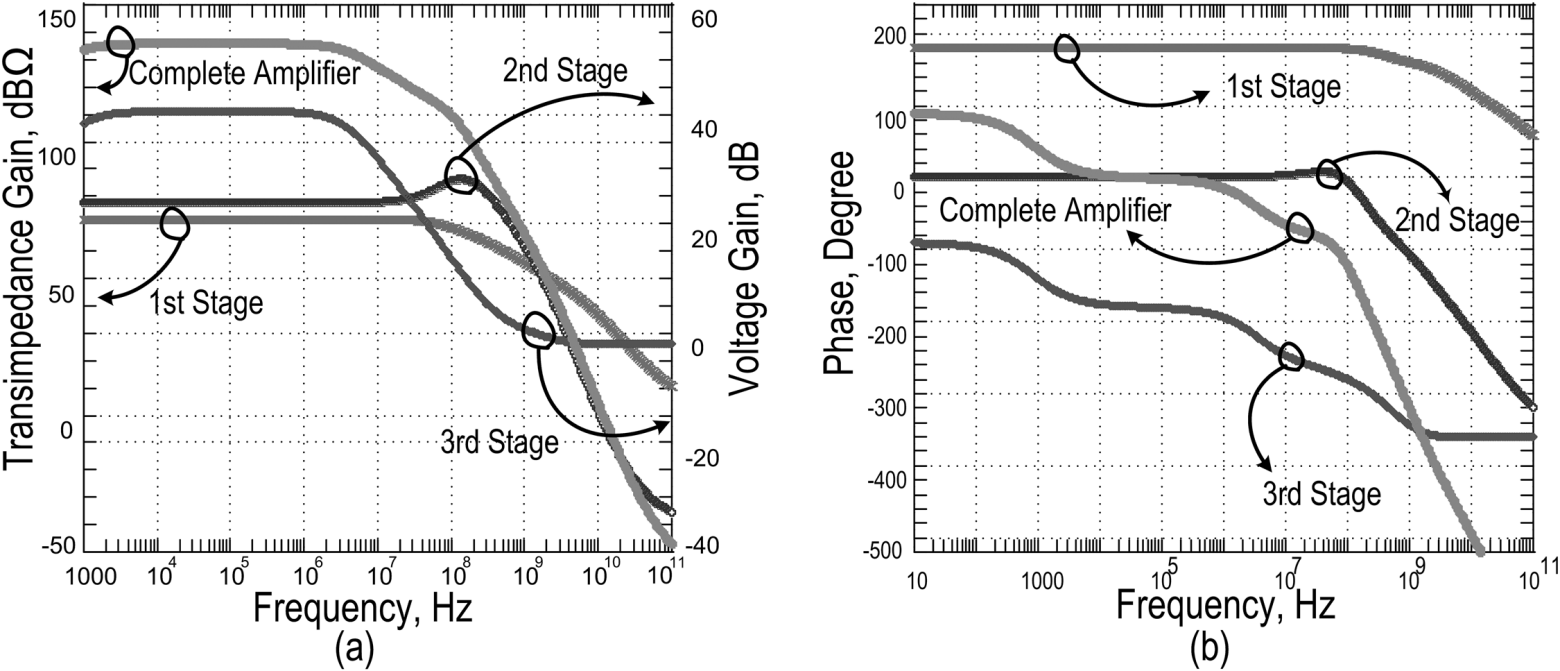
Open loop responses of sustaining amplifier. a) Gain plot of 1^st^ stage, 2^nd^ stage, 3^rd^ stage and complete amplifier b) Phase plots of 1^st^ stage, 2^nd^ stage, 3^rd^ stage and complete amplifier.

Fig 7 illustrates the responses from which a total open loop transimpedance gain is found at 122dBΩ with a phase shift of 70° at the operating frequency of 17.22MHz. Also, in Table 2, the corresponding gain and phase are summarized, contributing to this total transimpedance value.

**Table 2:** Open Loop Gain and Phases of the Different Stages Amplifier.

Amplifier stage	1^st^ stage: Transimpedance Amplifier	2^nd^ stage: Intr. Voltage Amplifier	3^rd^ stage: Cascode Amplifier	Complete Amplifier
**Gain**	72 dBΩ	24 dB	30 dB	122 dBΩ
**Phase (x°)**	182	5	-255	-70

**Fig 8:**
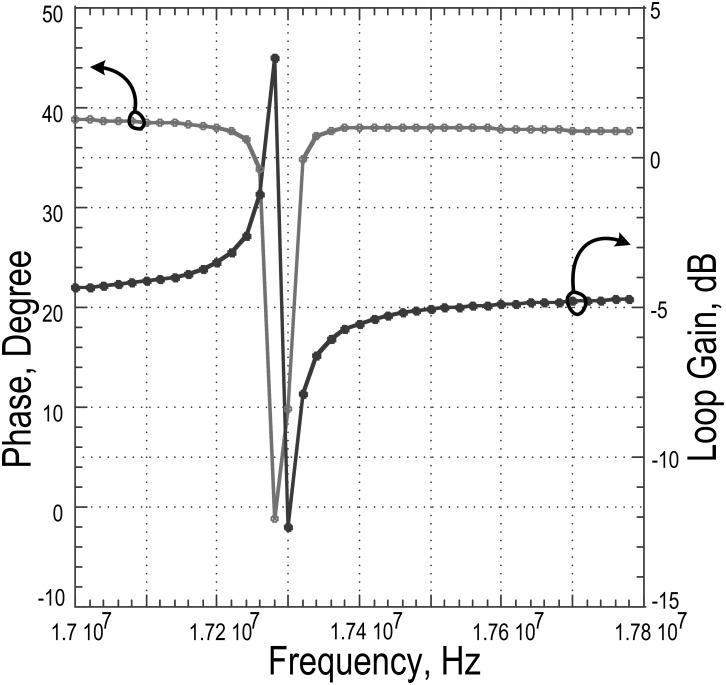
Loop gain and phase response for the complete oscillator.

To sustain the oscillation generated from the resonator model, the open loop gain must be above zero at the point of resonant frequency with the resonator included, confirming the Barkhausen criterion of oscillation [25]. The plot in Fig 8 illustrates the loop gain and phase shift at the operating frequency of 17.22MHz, which is observed to be 3.36dB and -1.12°, respectively.

#### Required TIA gain estimation

The insertion loss is estimated to be 76dB which is represented by motional *R*_*x*_ in Fig 6(a) for the C-C beam resonator, this loss ranges between several kΩ to a few MΩ and is dependent on the polarization voltage applied to it. While maintaining the linearity within the 90nm beam-electrode gap, for a bias of 5V, the reduced *R*_*x*_ is found to be 698kΩ in the present resonator. To sustain the oscillation in real time as quoted in F. Nabki’s work in [25], the effective motional resistance should be at least 1.5 times of *R*_*x*_, in dictating the minimum TIA gain. In this work, effective motional resistance is estimated to be *R*_*motional*_ ≥ 1.047MΩ. So, the minimum transimpedance gain that is adequate for sustaining the oscillation can be calculated as 20 log (1.047M) ≈ 120.398 dBΩ. With respect to that, the referred value of the transimpedance gain is 122dBΩ given in Table 2, which is well above this minimum threshold for sustaining the oscillation.

#### Sustaining amplifier open loop measurement result

**Fig 9:**
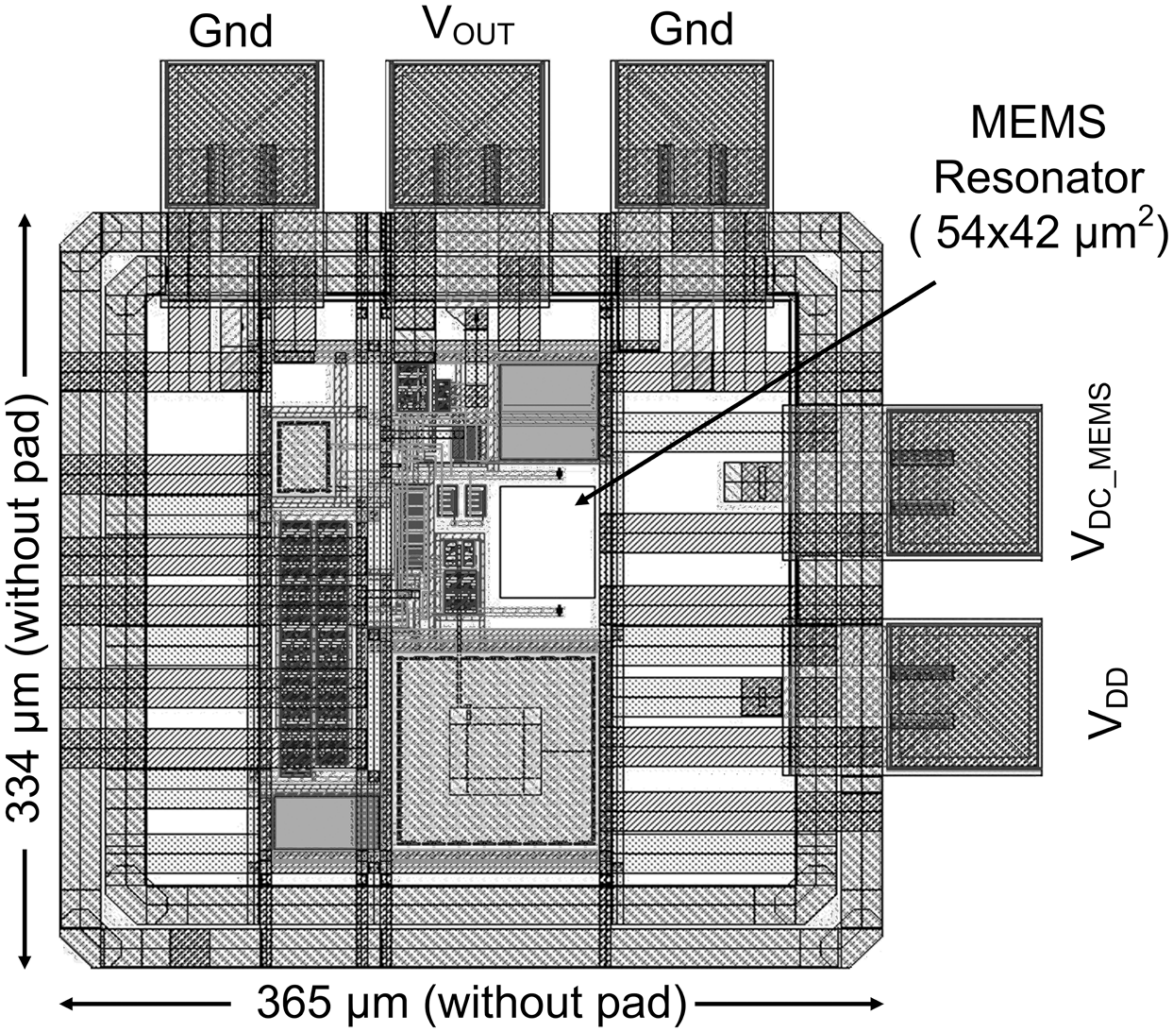
Complete layout of the quartz oscillator with the integrated MEMS resonator.

An open loop measurement set-up is used to obtain the DC bias point and current consumption from a 1.8V source for the designed amplifier. The area consumption of the complete oscillator architecture in the absence of the bond pads is 0.121mm^2^, as illustrated in Fig 9.

To obtain the forward transmission response of this sustaining amplifier, a -30dB_m_ of input power is applied to the RF_in_ port, to avoid saturation and the response is measured at RF_out_ with a 50Ω load termination.

**Fig 10:**
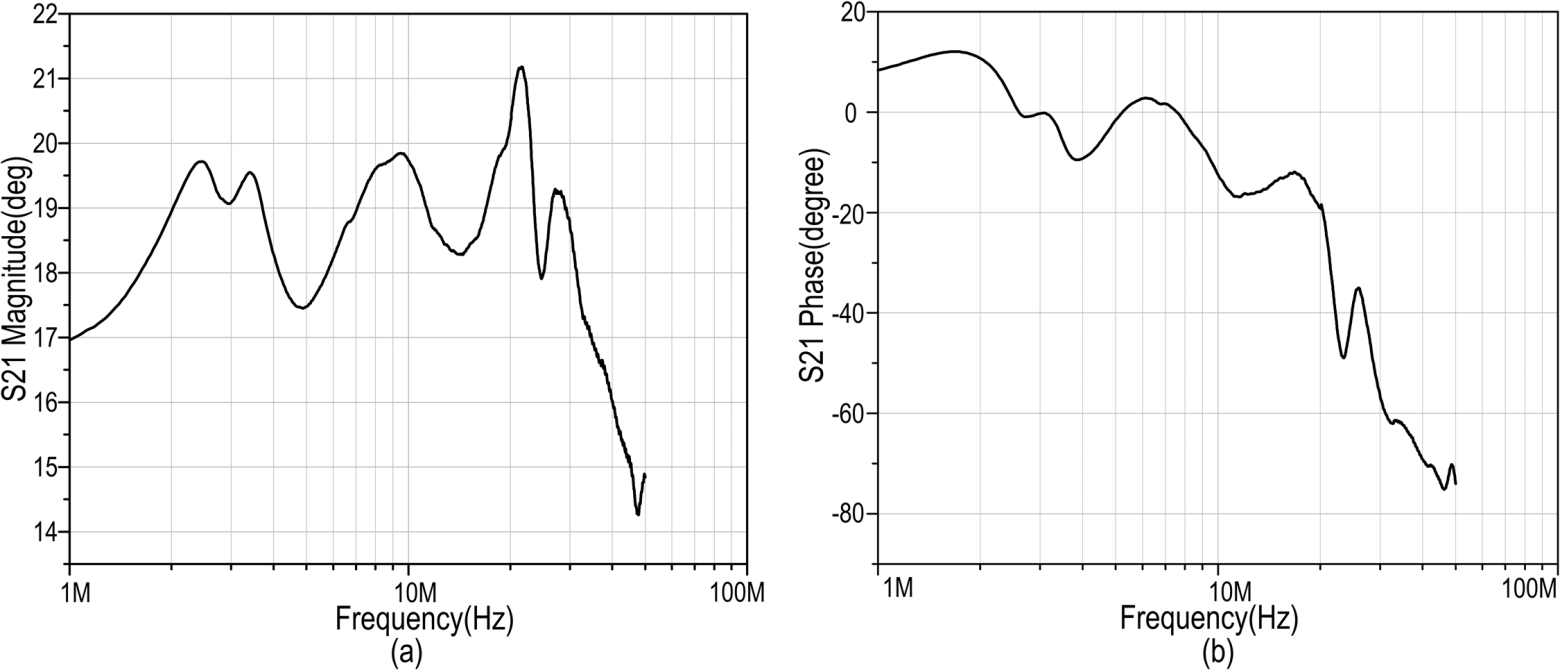
Measured forward transmission response of the sustaining amplifier chip. a) S_21_ gain plot b) S_21_ phase plot.

In regards to the 17.22MHz operational frequency, the measured scattering parameter responses are plotted in Fig 10 for frequency measurement in a sweep between 1MHz to 50MHz and with a IF bandwidth of 100Hz. As observed in Fig 10, a maximum gain of 21dB is achieved at 21MHz with a -3dB bandwidth of 40MHz which encapsulates the resonant frequency of the MEMS resonator. At the resonant frequency of 17.22MHz, the S_21_ magnitude is found as 19.48dB with a phase deviation of 12°. Hence, the loaded open loop gain at the resonant frequency, which is greater than 0dB ensures a sustained oscillation in closed loop configuration. This is also accompanied by the low phase shift of 12° at the resonant frequency which adequately satisfies the Barkhausen criteria.

### MEMS-oscillator close loop response and figure of merit

**Fig 11:**
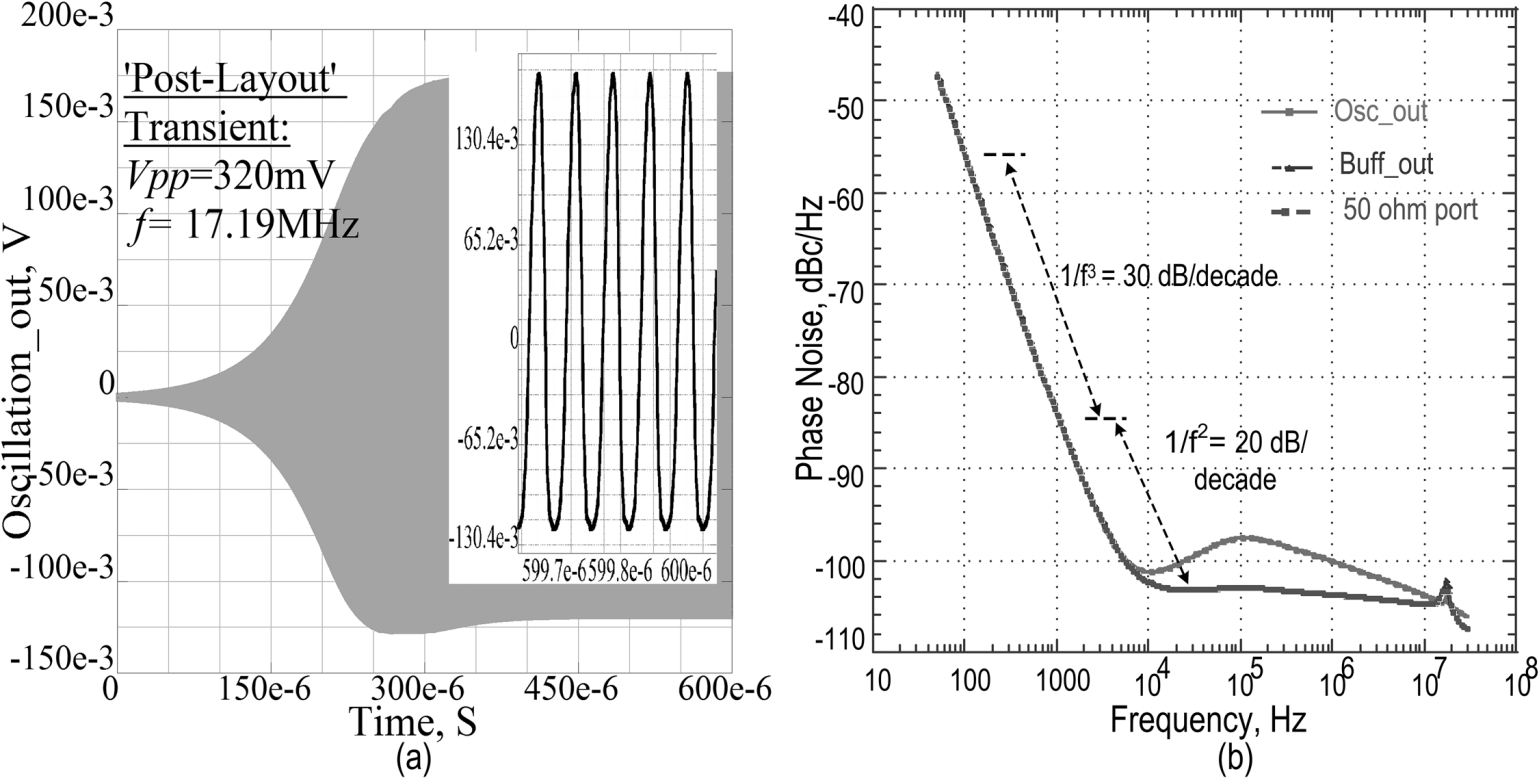
Post layout simulated close loop responses of the oscillator. a) Transient response at the measurement port (Inset is the magnified stable part of the oscillation) b) Phase noises at different points of sustaining amplifier.

In the close loop operation, the sustaining amplifier would provide the designed amount of gain to replenish all the motional losses. The unloaded quality factor of the MEMS device is only 1000 which is low enough to be competitive with the quartz counterpart and hence the phase noise measurement is crucial in its adaptation for an end-user specific application. In Fig 11, the phase noise spectrum is shown at reference points of the complete circuit architecture with the settling time of 450μs and a voltage swing of 320mV for transient response in the post layout simulation, the close to carrier phase noise is given in Fig 11(b) and observed to be 83.898dBc/Hz at 1kHz offset, whereas the far out level is 103.76dBc/Hz at 10MHz offset (Table 3). To guarantee the robustness of the proposed architecture, phase noise performance across different extreme corners and temperature is tabulated, as shown in Table 3.

**Table 3:** Phase Noise at Different extreme corners in Post Layout Simulation.

PN Point, dB_c_/Hz		@ 1kHz	@ 10kHz	@ 100kHz	@ 1MHz	@ 10MHz
Process	Temperature (°C)					
**All Typical**	27 (reference)	-83.9	-101.22	- 97.58	- 100.1	-103.76
	-25	-85.27	-102.13	-98.41	-101.14	-104.59
	125	-82.51	-99.92	-95.98	-98.84	-102.4
**All slow**	-25	-83.44	-100.17	-96.02	-100.3	-102.8
	125	-81.39	-98	-93.6	-97.91	-100.2
**All Fast**	-25	-86.01	-102.2	-98.8	-102.7	-104.98
	125	-83.93	-100.36	-96.02	-100.3	-102.7

The 1/*f*^3^ noise component, which dominates at 100Hz of offset frequency, consists of intermodulation in carrier noise and transistor noise component. In a low quality MEMS, the capacitive nonlinearity is the apparent contributor towards the flicker frequencies [26].

The phase noise expression in Eq (16), describes the dependency among the various parameters.

$$L\left\{f_{m}\right\}\approx \frac{kTF}{2V_{o}^{2}}\left[\frac{R_{x}}{Q_{l}^{2}}\right]\times {\left[\frac{C_{x}}{C}\right]}^{2}\times {\left[\frac{f_{0}}{f_{m}}\right]}^{2}$$ (16)

Here, *f*_*m*_ is the offset from the carrier frequency at which point phase noise is being evaluated, *V*_*o*_ is the oscillator output voltage magnitude, *C = C*_*l*_ is the external resonating capacitance, *k* is the Boltzmann’s constant, *Q*_*l*_ is the loaded quality factor of the tank, and *F* is the noise figure of the sustaining amplifier. A much closer inspection, reveals the post layout simulated phase noise response of the curve in Fig 11 is found to have a slope of 1/*f*^3^ at low frequency offsets. This can be predicted by inspecting Eq (16) thoroughly or by realizing the scaling-induced noise theories. With respect to the stability of this oscillator, phase noise of −83.898dBc/Hz at 1kHz offset from the carrier is a limiting factor, which may have been contributed by the following causes.

- Non-linearity in the resonator capacitive transducer aliases 1/*f*, electronic noise (e.g., from the sustaining amplifier) onto the carrier frequency, generating a 1/*f*^3^ component.
- 1/*f* noise associated with the DC polarization voltage on the resonator structure, which modulates the electrical stiffness *k* of the resonator [26, 27], inducing a 1/*f*^3^ phase noise component.
- 1/*f* mechanical noise which induces variations in the electrode-to-resonator gap spacing, which then modulates the electrical stiffness *k*, generating a 1/*f*^3^ phase noise component.

In reference to the oscillator application with the targeted Si micro-resonator, long term stability in material surface and noise performance are of significant concern among the above mentioned points where the limitation of non-linearity is the most likely mechanism, suspected to contribute towards additional noise being accounted in the phase noise spectrum [28] in achieving selectivity and sensitivity [26, 27]. The designed oscillator consumes 6.24mW of DC power, in the absence of the 50Ω buffer amplifier which was deliberately integrated for measurement purpose. In any clock IC application, there are other blocks, like fractional PLL, counter, etc. [29] for which the instrumentation buffer integration is alleviated.

**Fig 12:**
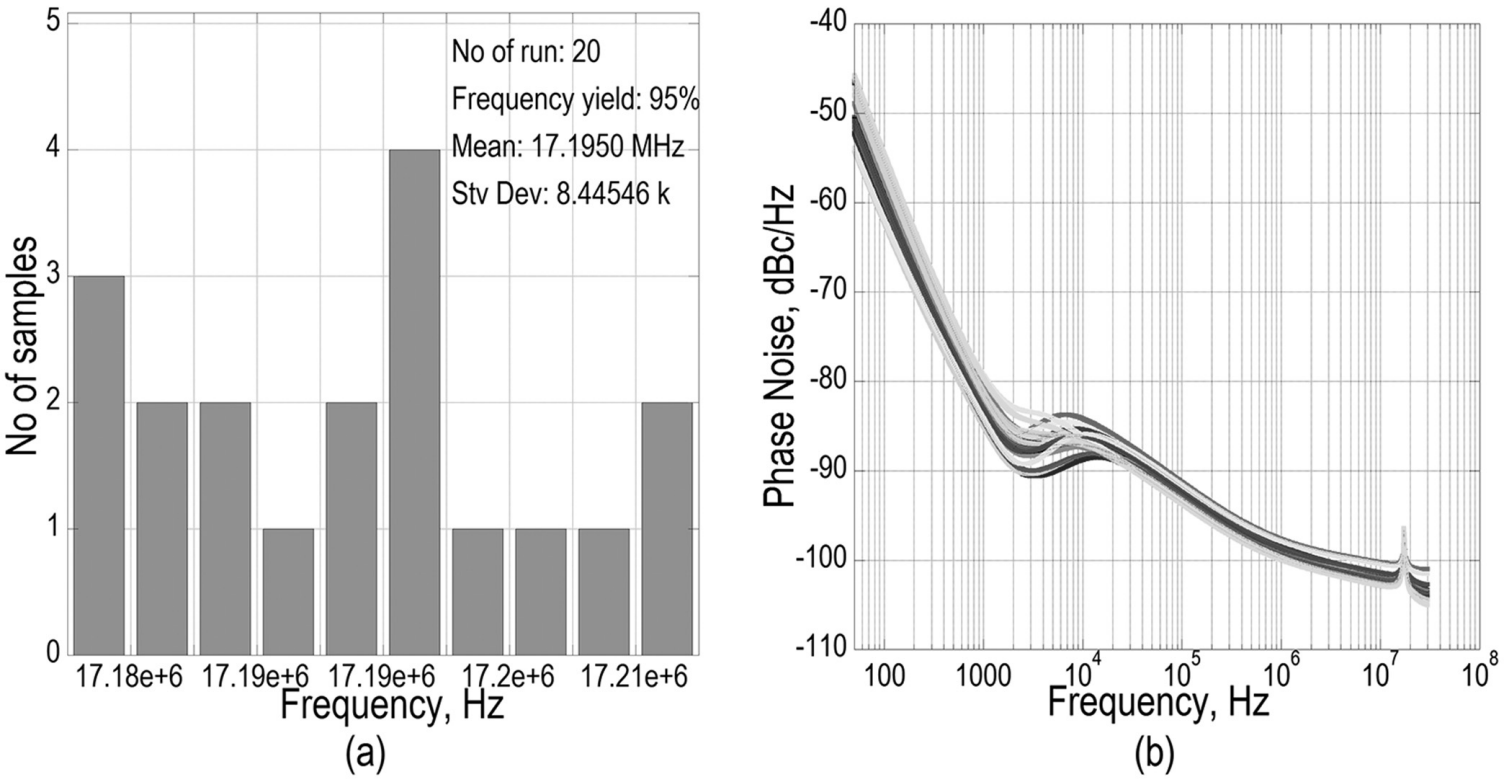
Monte Carlo post layout simulation results. a) Histogram plot for ‘Yield of Frequency’ at 50 Ω output port b) phase noise at 50 Ω output port for different run.

In practical realization of the presented work as a fixed frequency reference oscillator in clock IC application, a high percentage of frequency yield is targeted in statistical analysis. In Monte Carlo analysis regarding a random variation for process and mismatch, 95% frequency yieldin 20 number of run has been achieved [Fig 12(a)]. It is observable that the mean is closer to the desired frequency of oscillation of 17.22 MHz, which is supported by the tabulated data in Table 4.

**Table 4:** Monte Carlo Results for Oscillation Frequency at Different Nodes.

Node Name	Mean	Standard Deviation
**Cascade_stage_output**	17.1950 MHz	8.44546 k
**Buffer_amp_output**	17.1950 MHz	8.44546 k
**50 Ω port_output**	17.195 MHz	8.445 k

**Fig 13:**
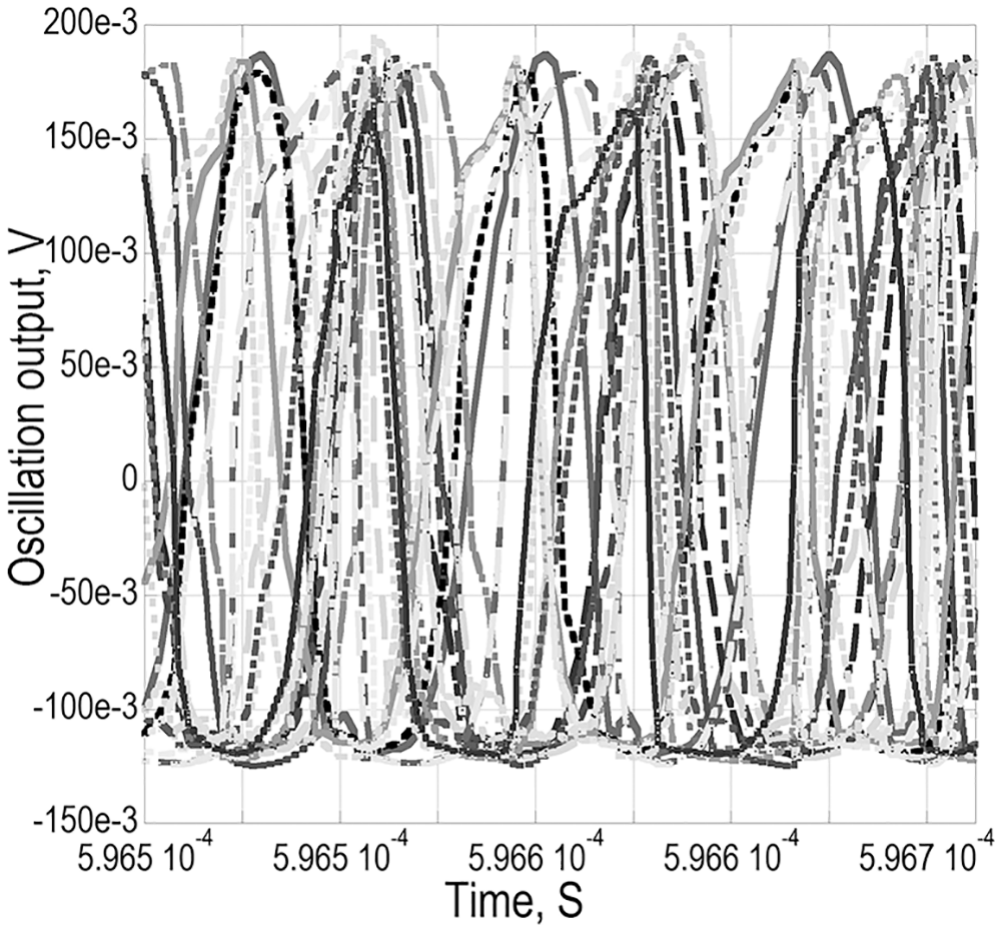
Transient response at 50 Ω output port in Monte Carlo simulation.

In Table 4, it is also observed that, the pad capacitance and buffer amplifier have no any additional ‘parasitics’ adversely affecting the frequency of oscillation. However, in the phase noise response seen in Fig 13, improvement in the 50 Ω measurement port is observed, as the buffer has added some additional gain without contributing into the noise power.

For the phase noise spectrum shown in Fig 12(b), noise floor converges at -104dB_c_/Hz in all the run of the MonteCarlo, but as seen in Fig 13, the amplitude slightly varies, which seems to be the resultant of TIA generated noise and its effect on the overall bandwidth of interest. The gain can be made more stable by extending the bandwidth a bit further which may improve the amplitude fluctuation seen in Fig 13, where in additional, an automatic gain control (AGC) can reflect this benefit as well. Now, the integration of AGC is avoided in this work in exploiting the merits in simplicity of design and less power consumption. In reference to the low unloaded *Q* of 1000 of the Si-MEMS, the achieved phase noise is acceptable for the synthesized oscillator targeted towards real time clock (RTC) application. Also, the intended MEMS architecture is a temperature uncompensated resonator which preserves a further improvement avenue for the oscillator response.

A Figure of Merit (FOM) is necessary to define all the degrading factors affecting the performance being computed. The defined FOM will be able to relate mechanical limitations with the electrical performance and the operating temperature, so that a competitive comparison can be tabulated among various *Q* based oscillator designs andit is given in Eq (17), developed on the basis of S. Seth *et al*. work on PLL [30].

$$F.O.M=\frac{1}{PN_{Floor}}\times \frac{kT}{P_{DC}}\times f_{o}^{2}\times R_{x}^{2}$$ (17)

Here, *k* is the Boltzman constant in J/kelvin, *T* is the temperature in kelvin, *P*_*DC*_ is the DC power consumption in watt, *f*_*o*_ is the operating frequency, *PN* is the phase noise floor and *R*_*x*_ is the motional resistance in Ω. In Table 5, the required comparison is shown where the FOM of this work has been found in an appreciating position as high as 0.94 Million, despite of its low *Q*, temperature uncompensated resonator or its lower range of operating frequency. As observed along with its possible monolithic integration, the reported MEMS oscillator can be adopted for a real time clocking IC application on the basis of the competitive results in Table 5.

**Table 5:** Design Parameters and Performance Comparison.

Parameter	Unit	[24]*^,^^†^	[4]*^,^^†^	[29]^†^	[14]^†^	[31]^†^	This work*^†^
**MEMS Resonator**							
Beam type	~~~	BAW	Tuning fork	T-shape	Nano-Plate	FBAR	Clamp-Clamp
Transduction Mode	~~~	Electrost-atic	Electrost-atic	Electrost-atic	Electrost-atic	Lateral field	Electrost-atic
Frequency	Hz	35.3 M	1.2 M	1 M	167.8 M	1.17 G	17.22 M
Motional Resistance	Ω	1.36k	700k	200	145	135	698k
Quality Factor	~~~~	1,800	3,029	20,000	1,084	2,200	1,000
Material	~~~~	AlN on Si	AlCu on Si	AlN on Si	AlN/FeGaB	AlN on Si	Si
**Sustaining Amplifier**							
Process	m	500 n	350 n	180 n	500 n	500 n	180 n
Supply Voltage	V	3	2.5	1	2.2	~~~~~	1.8
Transimpedance gain	dBΩ	83	126	~~~~~~~	~~~~~~	~~~~~	122
Configuration	~~~	Single	Single	Single	Single	Single	Single ended
Power consumption	W	3.8m	1.3m	3.2μ	3m	3.5m	6.24m (including buffer)
Layout area	mm^2^	1.96	0.052	0.1125	~~~~	~~~~~	0.121
**Oscillator**							
Type	~~~~	Monolith-ic	Monolith-ic	Monolith-ic	Wire bond	Monolith-ic	Monolith-ic
PN@1kHz	dBc/Hz	-108 (in HF mode)	-112	-70	-81.73	-81	-84
PN@10kHz	”	-130	~~~~~	-85	-110	-112	-97
Noise Floor	”	-140	-120	-110	-130	-146	-102
F.O.M	Hz^2^Ω^2^	18.13	18.72k	0.47	6.224	202.12	0.94M

* Post Simulation results.

^†^ Measurement results.

The compared works used AlN compounds in resonator part, resulting in better power handling capability through thermal compensation [31], [32]. Such advantage is absent in the proposed architecture, yet achieving the highest FOM. The mechanical *Q* of the used Si resonator is much lower than the *Q* of other compared works. This is reflected in higher motional losses, but the proposed sustained amplifier design has fully compensated the bigger losses. Operating frequency can be seen higher in [24], [29], [14] than the reported work of 17.22 MHz and still the design has achieved a comparable phase noise values. So, the current design is delivering a high FOM for the comparatively lower cost MEMS resonator in similarly categorized crystal oscillator application. This ensures a possible ‘low cost-high reliability’ penetration in the miniaturized clock IC markets for the realized design. Additionally, this work has used 180nm process technology compared to 500nm of [14], [24], [31], which means, this design has to suffer from higher leakage current originated problems, like higher static power consumption, random fluctuations, worse gradients, increased diffusion effects, etc. But, the higher FOM of the present work has overcome many of these problems and ensured a small form factor of the solution as well. Owing to the output buffer, the DC power consumption becomes relatively high. However, in practical applications, the buffer is not necessary.

## Conclusion

A monolithically implementable Si-CMOS reference oscillator has been reported in this work. A 17.22MHz series resonant C-C beam Si-MEMS device which is used as a resonator along with a sustaining amplifier and an output buffer has been designed in the 180nm CMOS process. The sustaining amplifier is designed to compensate the MEMS motional losses with its 122dBΩ gain and designated phase shift. Open loop simulation supports the performance of the sustaining amplifier where measureable results have defended it. In close loop, the CMOS circuitry is optimized in this oscillator for a phase noise performance of -84dBc/Hz at 1kHz of offset as required for an application in clock generator IC. The MEMS resonator operates in flexural mode of vibration to produce the capacitive current and hence has very limited quality factor, observed as low as 1000. The small form factor and comparable phase noise with a very high figure of merit has made the solution feasible for monolithic fabrication. Moreover, 95% yield in desired operative frequency of oscillation in Monte Carlo simulation response has defended the possible integration of the design in Si-CMOS process for a clock IC application.

## References

[1] Tabatabaei S, Partridge A. Silicon MEMS oscillators for high-speed digital systems. IEEE micro. 2010;(2):80–9.

[2] Ruffieux D, Scolari N, Giroud F, Le T-C, Dalla Piazza S, Staub F, et al A Versatile Timing Microsystem Based on Wafer-Level Packaged XTAL/BAW Resonators With Sub-W RTC Mode and Programmable HF Clocks. Solid-State Circuits, IEEE Journal of. 2014;49(1):212–22.

[3] Pachkawade V, Li M-H, Li C-S, Li S-S. A CMOS-MEMS Resonator Integrated System for Oscillator Application. Sensors Journal, IEEE. 2013;13(8):2882–9.

[4] Li M-H, Chen C-Y, Li C-S, Chin C-H, Li S-S. A monolithic CMOS-MEMS oscillator based on an ultra-low-power ovenized micromechanical resonator. Microelectromechanical Systems, Journal of. 2015;24(2):360–72.

[5] Ramiah H, Keat CW, Kanesan J. Design of Low-phase Noise, Low-power Ring Oscillator for OC-48 Application. IETE Journal of Research. 2012;58(5):425–8.

[6] Asl SZ, Mukherjee S, Chen W, Joo K, Palwai R, Arumugam N, et al, editors. 12.9 A 1.55× 0.85 mm 2 3ppm 1.0μA 32.768 kHz MEMS-based oscillator. Solid-State Circuits Conference Digest of Technical Papers (ISSCC), 2014 IEEE International; 2014: IEEE.

[7] Gronicz J, Pulkkinen M, Yücetaş M, Halonen K, editors. A 2μA temperature compensated mems-based real time clock with±4 ppm timekeeping accuracy. Circuits and Systems (ISCAS), 2014 IEEE International Symposium on; 2014: IEEE.

[8] Zaliasl S, Salvia JC, Hill GC, Chen LW, Joo K, Palwai R, et al A 3 ppm 1.5× 0.8 mm 2 1.0 μA 32.768 kHz MEMS-Based Oscillator. Solid-State Circuits, IEEE Journal of. 2015;50(1):291–302.

[9] Gong C-SA, Lin S-P, Mandell MS, Tsou M-Y, Chang Y, Ting C-K. Portable Optical Epidural Needle-A CMOS-Based System Solution and Its Circuit Design. PLoS ONE. 2014;9(8):e106055 doi: 10.1371/journal.pone.0106055 25162150 10.1371/journal.pone.0106055 PMC4146568

[10] Bhugra H, Lee S, Pan W, Pai M, Lei D, editors. Commercialization of world's first piezomems resonators for high performance timing applications. Micro Electro Mechanical Systems (MEMS), 2014 IEEE 27th International Conference on; 2014: IEEE.

[11] Thakar VA, Wu Z, Peczalski A, Rais-Zadeh M. Piezoelectrically transduced temperature-compensated flexural-mode silicon resonators. Microelectromechanical Systems, Journal of. 2013;22(3):815–23.

[12] Poddar AK, Rohde UL. Latest Technology, Technological Challenges, and Market Trends for Frequency Generating and Timing Devices [Application Notes]. Microwave Magazine, IEEE. 2012;13(6):120–34.

[13] Langfelder G, Caspani A, Tocchio A. Design criteria of low-power oscillators for consumer-grade MEMS resonant sensors. Industrial Electronics, IEEE Transactions on. 2014;61(1):567–74.

[14] Hui Y, Nan T, Sun NX, Rinaldi M. High resolution magnetometer based on a high frequency magnetoelectric MEMS-CMOS oscillator. Microelectromechanical Systems, Journal of. 2015;24(1):134–43.

[15] Sobreviela G, Uranga A, Barniol N, editors. Tunable transimpedance sustaining-amplifier for high impedance CMOS-MEMS resonators. Microelectronics and Electronics (PRIME), 2014 10th Conference on Ph D Research in; 2014: IEEE.

[16] Li M-H, Chen C-Y, Li S-S, editors. An experimental investigation on the Q-boosted CMOS-MEMS flexural-mode resonator circuits. Frequency Control Symposium (FCS), 2014 IEEE International; 2014: IEEE.

[17] Sobreviela G, Riverola M, Uranga A, Barniol N, editors. Noise effects on resonator bias polarization in CMOS-MEMS oscillators. SENSORS, 2014 IEEE; 2014: IEEE.

[18] Arefin MS, Redoute J-M, Yuce MR. A MEMS Interface IC With Low-Power and Wide-Range Frequency-to-Voltage Converter for Biomedical Applications. 10.1109/TBCAS.2015.2435256 26954843

[19] Sedra AS, Smith KC. Microelectronic circuits: New York: Oxford University Press; 1998.

[20] Phang K. CMOS optical preamplifier design using graphical circuit analysis: University of Toronto; 2001.

[21] Razavi B. Design of integrated circuits for optical communications: John Wiley & Sons; 2012.

[22] Verd J, Uranga A, Abadal G, Teva J, Torres F, Lopez J, et al Monolithic CMOS MEMS oscillator circuit for sensing in the attogram range. Electron Device Letters, IEEE. 2008;29(2):146–8.

[23] Lavasani HM, Pan W, Harrington B, Abdolvand R, Ayazi F. A 76 dB 1.7 GHz 0.18 m CMOS tunable TIA using broadband current pre-amplifier for high frequency lateral MEMS oscillators. Solid-State Circuits, IEEE Journal of. 2011;46(1):224–35.

[24] Lavasani HM, Abdolvand R, Ayazi F. Single-resonator dual-frequency AIN-on-Si MEMS oscillators. Ultrasonics, Ferroelectrics, and Frequency Control, IEEE Transactions on. 2015;62(5):802–13. 10.1109/TUFFC.2015.007051 25965675

[25] Nabki F, Allidina K, Ahmad F, Cicek P-V, El-Gamal MN. A highly integrated 1.8 GHz frequency synthesizer based on a MEMS resonator. Solid-State Circuits, IEEE Journal of. 2009;44(8):2154–68.

[26] Pardo M, Sorenson L, Ayazi F. An empirical phase-noise model for MEMS oscillators operating in nonlinear regime. Circuits and Systems I: Regular Papers, IEEE Transactions on. 2012;59(5):979–88.

[27] He L, Xu YP, Palaniapan M. A state-space phase-noise model for nonlinear MEMS oscillators employing automatic amplitude control. Circuits and Systems I: Regular Papers, IEEE Transactions on. 2010;57(1):189–99.

[28] Razavi B. A study of phase noise in CMOS oscillators. Solid-State Circuits, IEEE Journal of. 1996;31(3):331–43.

[29] Ruffieux D, Krummenacher F, Pezous A, Spinola-Durante G. Silicon resonator based 3.2 W real time clock with 10 ppm frequency accuracy. Solid-State Circuits, IEEE Journal of. 2010;45(1):224–34.

[30] Seth S, Wang S, Kenny T, Murmann B, editors. A− 131-dBc/Hz, 20-MHz MEMS oscillator with a 6.9-mW, 69-kΩ, gain-tunable CMOS TIA. ESSCIRC (ESSCIRC), 2012 Proceedings of the; 2012: IEEE.

[31] Zuo C, Van der Spiegel J, Piazza G. 1.05-GHz CMOS oscillator based on lateral-field-excited piezoelectric AlN contour-mode MEMS resonators. Ultrasonics, Ferroelectrics, and Frequency Control, IEEE Transactions on. 2010;57(1):82–7. 10.1109/TUFFC.1382 20040430

[32] Zuo C, Rinaldi M, Piazza G. Power handling and related frequency scaling advantages in piezoelectric AlN contour-mode MEMS resonators. 2009.

